# Stress and burnout among Greek critical care nurses during the COVID-19 pandemic

**DOI:** 10.3934/publichealth.2023051

**Published:** 2023-09-07

**Authors:** Thiresia Sikioti, Afroditi Zartaloudi, Despoina Pappa, Polyxeni Mangoulia, Evangelos C. Fradelos, Freideriki Eleni Kourti, Ioannis Koutelekos, Evangelos Dousis, Nikoletta Margari, Areti Stavropoulou, Eleni Evangelou, Chrysoula Dafogianni

**Affiliations:** 1 Hellenic Open University, Athens, Greece; 2 Faculty of Nursing, University of West Attica, Athens, Greece; 3 Faculty of Nursing, National and Kapodistrian University of Athens, Athens, Greece; 4 Faculty of Nursing, University of Thessaly, Greece; 5 Medical School, National and Kapodistrian University of Athens, Athens, Greece

**Keywords:** job stress, burnout, intensive care unit (ICU), nurses, COVID-19, resilience

## Abstract

Occupational stress and burnout of health personnel during the COVID-19 pandemic, especially of the nursing population in intensive care units (ICUs), were quite frequent along with negative effects and a direct correlation with the manifestation of many physical, behavioral and psychological symptoms. For the purposes of this research, a quantitative survey was carried out, in which 153 ICU nurses of secondary and tertiary public hospitals in Greece participated. Nurses completed anonymously and voluntarily a special electronic questionnaire about stress, burnout, personal concerns about the pandemic, the consequences of the outbreak and their resilience toward COVID-19 patients' care. Specific validated scales were used in this study. Female nurses felt, to a greater extent than males, work-related burnout, especially patient-related burnout and total burnout. There was a statistically significant negative relationship between the existence of a psychological support group within a hospital and personal burnout. Participants who had experience in caring for SARS-CoV-2 patients had higher mental resilience than those without experience. As the consequences experienced by the health professionals of the reference COVID-19 hospitals were increased, so did mental resilience and stress coping strategies during the pandemic. The COVID-19 outbreak and the conditions configurated in the health system had negative effects on the psycho-emotional state of ICU nurses. The manifestation of anxiety, stress and burnout had a direct correlation with both the work and personal functionality of the nurses and the whole of the healthcare services provided. The early recognition of symptoms and their individualized management are imperative for the protection of the psycho-emotional well-being of nurses.

## 1. Introduction

In February 2020, COVID-19, an outbreak of a new coronavirus disease, was declared a Public Health Emergency of International Concern by the World Health Organization (WHO) [1]. WHO determined in March 2020 that COVID-19 can be considered a pandemic [2]. The large and ongoing stream of contaminated patients put hospitals, and especially intensive care units (ICUs), under extreme stress. Within a few weeks after the pandemic's start, the ICUs became overcrowded, creating a critical need for more beds and additional resources to treat the patients [3]. Healthcare professionals (HCPs) experienced unprecedented levels of uncertainty and instability as a result of the COVID crisis, and they were completely worn out from stress.

Before the outbreak, specialists had described the intensive care unit's working environment as demanding, particularly because of ongoing technological advancement, the difficulties associated with end-of-life care, problems with organ retrieval, heavy workload and night shifts [4],[5]. According to Mangoulia et al. [6],[7], 61.5% of the ICU nurses they surveyed indicated a limited potential for compassion satisfaction, while 57.9% of ICU nurses experience an increased risk for secondary traumatic stress / compassion fatigue (STS/CF), and 56.1% are at a high level of risk for burnout. The risk of developing compassion fatigue was shown to be higher in female nurses, low-income individuals, married or widowed individuals and assistant nurses. Additionally, it was found that burnout has a high positive link with compassion fatigue and a strong negative relationship with compassion satisfaction. These burdens worsen HCPs' health-related quality of life by impairing their caring behaviors, increasing errors and cost of caring and worsening health outcomes and productivity (presenteeism and absenteeism) [8].

The COVID-19 pandemic has completely disrupted people's daily lives, commerce, the educational system and the economy [9]. This critical situation puts on the front line HCPs who are directly involved in the diagnosis, treatment and care for patients with the most severe cases of the disease [10]. During medical examinations and in the operating rooms, ICU HCPs may be more susceptible to psychological discomfort. They participate in aerosol-generating medical procedures and watch the most severe cases of disease, increasing the risk of infection transmission [11].

The fear of infection and spreading it to family and friends, the heavy workload, the sporadic lack of personal protective equipment and the requirement to take strenuous precautions during the medical examination and in the operating rooms can all significantly increase the psychological burdens on HCPs [12],[13]. According to research by Moore et al. [14] in the United States that looked at ICU nurses' experiences during the COVID-19 outbreak, they felt uneasy and afraid for their lives while caring for COVID-19 patients.

Although it can be rewarding for nurses to help patients who are going through severely difficult situations, the stress involved in doing so can have a negative effect on the nurses themselves [8]. According to studies, some ICU nurses have suffered from somatic and psychological problems, like stress, sadness, anxiety, post-traumatic stress disorder and peritraumatic dissociation as a result of the COVID-19 outbreak [15],[16], as well as reduced appetite or dyspepsia, nervousness, fatigue, sleeping disorders, repeated crying and, in some extreme cases, thoughts of suicide [17]. Significant links between insomnia, drowsiness, depression and job stress were found [18].

Because of how hard the COVID ICU work was, nurses did not have the stamina to be as close to their families as they wanted. That was identified as the conscience's most trying quality [19]. Resilience is the most important coping strategy used by ICU nurses [20]. The main causes of occupational stress were long work hours, high nurse-to-patient ratios, a heavy workload, and poor patient outcomes. Gender, the number of children, the number of years spent in intensive care and the type of work shifts all had impacts on occupational stress [21].

The aim of this study was to identify the sources of stress and burnout among ICU nurses during the COVID-19 pandemic. The results of this study can aid in the creation of methods for reducing the stress experienced by ICU nurses. The prevention of mental illness and the protection of nurses' psycho-emotional well-being, whose maintenance requires interventions on personal as well as institutional and organizational levels, prioritize the early recognition of symptoms and their individual care.

## 2. Materials and methods

### 2.1. Sample and data

The population of the study consisted of 153 nurses of intensive care units in secondary and tertiary Greek public hospitals. The survey was conducted between November 2021 and February 2022 after approval from the Hellenic Open University's Ethics Committee (52654–20/07/2020). ICU nurses from 18 hospitals were informed about the purpose, the necessity and anonymity of the research and completed the online questionnaire after consenting at the beginning of the research tool via the specific “I Agree” option. Hospitals that gave permission to conduct the survey were included in the research.

### 2.2. Measure of variables

The research tool was provided electronically to the participants due to the restrictive measures applied during the outbreak. The questionnaire used consisted of 6 parts. The first part included demographic questions. The second section of the questionnaire examined the nurses' burnout in ICUs. The Copenhagen Burnout Inventory (CBI) is a 19-item self-reported measure of burnout. It contains three sub-scales measuring personal burnout, work-related burnout and client-related burnout. It consists of 19 questions on a 5-point Likert scale [22]. The third part of the questionnaire included the Expanded Nurse Stress Scale (ENSS) investigating the stress of nurses and consisting of 57 questions on a 6-point Likert scale. The fourth part of the questionnaire examined the consequences experienced by healthcare professionals of the COVID-19 reference hospitals [23],[24]. The fifth part of the questionnaire, which referred to the Brief Resilience Scale (BRS) [25], consisted of 6 statements. The Brief Resilience Scale was created to assess the perceived ability to bounce back or recover from stress. The scale was developed to assess a unitary construct of resilience, including both positively and negatively worded items. The possible score range on the BRS is from 1 (low resilience) to 5 (high resilience). The response scale of this section is Likert (1: Strongly Disagree – 5: Strongly Agree). Finally, the sixth part of the questionnaire (BriefCope) assessed the coping strategies toward stress during the period of the COVID-19 pandemic [26],[27].

### 2.3. Data analysis

The study data were analyzed with the SPSS statistical package to describe the variables and identify correlations and differences. Frequencies and averages were used to describe the variables, while independent samples t-test, ANOVA, Pearson correlation and multiple regression statistical analyses were used for correlations and differences. The normality of the distributions of the scales was checked via Kolmogorov-Smirnov tests.

## 3. Results

Ultimately, 153 nurses working in tertiary and secondary Greek hospital ICUs were included in the study. The questionnaire was initially distributed to 190 ICU nurses. Μost participants were female (N = 135, 88.2%), and 11.8% of them were male (Table 1). Also, participant ages ranged from 24–58 years. The mean age of the participants was approximately 37 years with a standard deviation of 7.9 years. Almost half of the sample had children (N = 77, 50.3%). Μost participants were graduates of Technological Education Institutes (N = 91, 59.5%). Furthermore, 24.2% of the participants held a master's degree, 8.5% of them were university graduates, and 7.8% of them were secondary school graduates. Of the participants, 33.3% had 1–5 years of experience in a nursing job. 24.2% had 11–15 years of experience, 19.6% had 16–20 years, 12.4% had 6–10 years of experience, and 10.4% 21 years or more of nursing experience.

Almost all participants mentioned normal working hours (N = 152, 99.3%). Specifically, 0.7% of them had a maternity work program. 90.2% of the sample worked in cyclical working hours. 7.8% worked morning shifts, with 1.3% on afternoon shifts and 0.7% of them on night shifts. 87.6% of the sample had experience in caring for patients with SARS-CoV-2, and 12.4% of them did not. 60.1% of the sample had received training in the care of patients with SARS-CoV-2. 51.6% of the sample mentioned that they did not have psychological support in the hospital they worked at. Furthermore, most participants had not sought individual psychological support outside their workplace during the COVID-19 pandemic period (N = 128, 83.7%), while 16.3% of them did seek individual psychological support outside their workplaces during that period.

**Table 1.** Sample characteristics.

Project	N (%)
Gender	
Men	18 (11.76%)
Women	135 (88.24%)
Married	67 (36.60%)
Not married	75 (48.37%)
Divorced	10 (6.54%)
Living with partner	1 (0.65%)
Children	
Yes	77 (50.33%)
No	76 (49.67%)
Educational level	
2-year college graduate	4 (7.84%)
University alumni	96 (67.98%)
MSc/PhD holder	53 (24.18%)
Years of experience in present hospital, median (IQR)	12 (1–30+)
Rotation shifts	147 (90.20%)
Morning shifts	3 (7.84%)
Evening shifts	2 (1.31%)
Night shifts	1 (0.65%)

### 3.1. Professional burnout

The main descriptive elements of the occupational burnout of the nurses in the ICUs during the COVID-19 outbreak were personal burnout (*Mean* = 20.5, *SD* = 5.27), work-related burnout (*Mean* = 23, *SD* = 5.19) and patient-related burnout (*Mean* = 17.9, *SD* = 5.33). Finally, total burnout was also measured (*Mean* = 61.5, *SD* = 14.77).

The t-test between participants' gender and work-related burnout (Table 2) revealed that there were statistically significant differences between genders with respect to personal burnout (as the significance values were set at 95%) (*t*151 = -5.312, *p* < 0.005), work-related burnout (*t*31.3 = -6.453, *p* < 0.005), patient-related burnout (*t*151 = -3.799, *p* < 0.005) and total burnout (*t*151 = -4.851, *p* < 0.005). More specifically, women experienced to a greater extent than men personal burnout, work-related burnout, patient-related burnout and total burnout.

Table 3 presents the t-test between participants' SARS-CoV-2 patient care experience and participants' work-related burnout. The following table shows that there is no statistically significant difference between the experience of caring for patients with SARS-CoV-2 and the work-related burnout of the participants, since the level of significance is greater than 0.05.

There were statistically significant differences between the existence of a psychological support group in the hospital and personal burnout (*t*151 = -3.354, *p* < 0.005), work-related burnout (*t*151 = -2.205, *p* < 0.005), patient-related burnout (*t*151 = -2.865, *p* < 0.005) and total burnout (*t*151 = -3.010, *p* < 0.005). More specifically, participants who did not have a psychological support group in the hospital feel to a greater extent, than those who did, personal burnout, work-related burnout, patient-related burnout and total burnout.

**Table 2.** The t-test between participants' gender and work-related burnout.

Project	Gender	N	*Μean*	*SD*	*t*	df	sig
Personal burnout	Male	18	14.8	3.49	-	151	*p* < 0.001
	Female	135	21.3	5.00	5.312		
Work-related burnout	Male	18	18.2	3.06	-	31.3	*p* < 0.001
	Female	135	23.7	5.09	6.453		
Patient-related burnout	Male	18	13.6	5.45	-	151	*p* < 0.001
	Female	135	18.5	5.07	3.799		
Total burnout (CBI)	Male	18	46.7	11.16	-	151	*p* < 0.001
	Female	135	63.4	14.08	4.851		

Note: The null hypothesis that was tested was that mean values for men and women were equal *vs*. the alternative hypothesis that they were different.

**Table 3.** t-test between participants' SARS-CoV-2 patient care experience and participants' work-related burnout.

Project	Do you have experience in SARS-CoV-2 patient care?	N	*Mean*	*SD*	*t*	df	sig
Personal burnout	Yes	134	20.6	5.26	0.426	151	0.670
	No	19	20.1	5.42			
Work-related burnout	Yes	134	23.1	5.19	0.730	151	0.466
	No	19	22.2	5.31			
Patient-related burnout	Yes	134	18.1	5.44	1.024	151	0.307
	No	19	16.7	4.5			
Total burnout (CBI)	Yes	134	61.8	14.8	0.778	151	0.438
	No	19	59.0	14.			

Note: The null hypothesis that was tested was that mean values for men and women were equal *vs*. the alternative hypothesis that they were different.

From the ANOVA test between marital status and work-related burnout of the nurses, it was revealed that there was a statistically significant difference between the marital status of the participants and personal burnout F(4, 152) = 3.016, *p* < 0.05 and total burnout F(4, 152) = 2.729, *p* < 0.05. The hypothesis tested was that means were equal across all marital statuses versus the alternative that at least one means differed from the others. More specifically, the participants who were married felt a greater degree of personal burnout and total burnout, compared to the single ones.

ANOVA test between the years of previous service as a nurse and the work-related burnout of the participants showed that there were statistically significant differences between the years of previous service as a nurse of the participants and personal burnout F(6, 152) = 4,573, *p* < 0.05, work-related burnout F(6, 152) = 4.920, *p* < 0.05, patient-related burnout F(6, 152) = 5.471, *p* < 0.05 and total burnout F(6, 152) = 5.520, *p* < 0.05. The hypothesis tested was that means were equal across all groups of years of previous service as a nurse versus the alternative that at least one mean differed from the others. More specifically, participants with 11–15 years of experience experienced more personal burnout, work-related burnout, patient-related burnout and total burnout than those with either 1–5 years of experience or 26–30 years. ANOVA test between the working shift with hours and the participants' work-related burnout showed, also, that there was no statistically significant difference between the participants' work shifts and burnout, since the level of significance was greater than 0.05.

### 3.2. Measurement and assessment of nursing stress in intensive care units

In the present study, it appeared that the participants were often stressed about their contact with death (*Mean* = 21.7, *SD* = 4.81), insufficient preparation to handle the emotional needs of patients and families (*Mean* = 9.4, *SD* =2.58) and discrimination (*Mean* = 9.3, *SD* =2.84). Also, from the following table it appears that the participants were often stressed about the workload (*Mean* = 25.7, *SD* = 5.80), about the uncertainty about the therapeutic result (*Mean* = 29, *SD* = 6.90) and about disputes with doctors (*Mean* = 15.4, *SD* = 4.00). Finally, participants were often stressed about conflicts with their colleagues (*Mean* = 19.8, *SD* = 5.25), about conflicts with superiors (*Mean* = 21.6, *SD* = 5.33) and about disputes with patients and their families (*Mean* = 23, *SD* = 5.77).

Table 4 shows the *t*-test between the gender of the participants and the measurement and assessment of nursing stress in intensive care units. There was no difference between the gender of the participants in the measurement and assessment of nursing stress in intensive care units, since the level of significance was greater than 0.05.

In order to check if ENSS scales were associated with participants' having experience in caring for SARS-CoV-2 patients, with receiving training in the care of SARS-CoV-2 patients and with searching for individual psychological support outside the workplace of the nurses during the COVID-19 outbreak, independent samples t-tests were used. There was a statistically significant difference between having experience in caring for SARS-CoV-2 patients of the participants and uncertainty about the therapeutic effect (*t*151 = 1.191, *p* < 0.05). Also, a statistically significant difference appeared between receiving training in the care of SARS-CoV-2 patients of the participants and uncertainty about the therapeutic effect (*t*151 = 1.191, *p* < 0.05).

In this study, there was a statistically significant difference between the search for individual psychological support outside the workplace of the nurses during the COVID-19 outbreak and the insufficient preparation to handle the emotional needs of patients and their families (*t*60.2 = 2.181, *p* < 0.05), workload (*t*151 = 2.229, *p* < 0.05), uncertainty about the therapeutic effect (*t*51.6 = 2.177, *p* < 0.05), disputes with doctors (*t*151 = 2.037, *p* < 0.05), conflicts with colleagues (*t*60.3 = 2.235, *p* < 0.05) and conflicts with patients and their families (*t*51.8 = 3.283, *p* < 0.05).

**Table 4.** The t-test between the gender of the participants and the measurement and assessment of nursing stress (ENSS) in intensive care units.

Stressor	Gender	N	*Mean*	*SD*	*t*	df	sig
Patient death	Male	18	21.0	6.12	-0.693	151	0.490
	Female	135	21.8	4.63			
Insufficient preparation of patients and families' needs management	Male	18	9.1	3.22	-0.469	151	0.640
	Female	135	9.4	2.49			
Racism	Male	18	9.6	3.35	0.362	151	0.718
	Female	135	9.3	2.78			
Workload	Male	18	25.4	8.42	-0.277	151	0.783
	Female	135	25.8	5.40			
Uncertainty about therapeutic effect	Male	18	28.7	7.88	-0.235	151	0.815
	Female	135	29.1	6.79			
Conflicts with doctors	Male	18	15.2	4.64	-0.242	151	0.809
	Female	135	15.4	3.88			
Conflicts with workmates	Male	18	17.3	6.01	-2.161	151	0.072
	Female	135	20.1	5.08			
Conflicts with superiors	Male	18	22.5	7.93	0.732	151	0.465
	Female	135	21.5	4.92			
Conflicts with patients and their families	Male	18	20.9	6.90	-1.595	151	0.112
	Female	135	23.2	5.57			

Note: The null hypothesis that was tested was that mean values for men and women were equal *vs*. the alternative hypothesis that they were different.

### 3.3. The consequences experienced by the health professionals of the COVID-19 referral hospitals

It appears that the participants several times felt discomfort caused by the protective equipment (*Mean* = 23.7, *SD* = 5.82). They experienced anxiety and difficulties related to the control of transmission and infection (*Mean* = 21.1, *SD* = 4.72) and felt quite the burden of patient care (*Mean* = 22.5, *SD* = 5.78). The following table (Table 5) shows that there was a difference between the genders of the participants and the discomfort caused by protective equipment (*t*151 = -3.400, *p* < 0.05) and the burden of patient care (*t*32.2 = -2.773, *p* < 0.05). More specifically, women felt a greater degree of discomfort caused by protective equipment and the burden of patient care than men.

**Table 5.** The t-test between gender of participants and consequences experienced by healthcare professionals of reference hospitals of COVID-19

Stressor	Gender	N	*Mean*	*SD*	*t*	df	sig
Discomfort caused by protective equipment	Male	18	19.5	5.02	-3.400	151	0.001
	Female	135	24.3	5.70			
Contamination and infection-related anxiety and difficulties	Male	18	20.1	4.82	-0.967	151	0.335
	Female	135	21.2	4.70			
Burden of patient care	Male	18	20.2	3.49	-1.867	151	0.009
	Female	135	22.9	5.96			
Concern related to working in the Hospital during COVID-19	Male	18	28.6	5.77	0.150	151	0.881
	Female	135	28.3	5.50			
Concern and worry outside of work in relation to COVID-19	Male	18	39.8	7.12	1.440	151	0.152
	Female	135	36.8	8.48			
Impact on personal life and work due to COVID-19	Male	18	39.4	8.40	-1.577	151	0.117
	Female	135	43.4	10.34			
Pandemic preparedness	Male	18	40.9	11.08	-0.567	151	0.571
	Female	135	42.4	9.76			

Note: 1. The null hypothesis that was tested was that mean values for men and women were equal vs the alternative hypothesis that they were different. 2. Greater scores indicate greater consequences.

There was a difference between receiving training in the care of patients with SARS-CoV-2 and the degree of agreement of the participants regarding pandemic preparedness (*t*151 = 4.173, *p* < 0.05). Furthermore, there was a statistically significant difference between the level of education and the degree of agreement for reflection in relation to work in the hospital and COVID-19 F(4, 152) = 4.746, *p* < 0.05, for reflection and worry outside of work due to COVID-19 F(4, 152) = 6.007, *p* < 0.05 and for the impact on personal life and work due to COVID-19 F(4, 152) = 3.492, *p* < 0.05. More specifically, the participants who were Technological Education Institute graduates agreed to a greater extent about the reflection in relation to work in the hospital and COVID-19 and the reflection and worry outside of work due to COVID-19, in relation to the university graduates and those with a master's degree. Also, participants who were university graduates agreed to a lesser extent about the effects on personal life and work due to COVID-19, than secondary education and Technological Education Institute graduates.

ANOVA test between years of service and the consequences experienced by health professionals of the reference hospitals COVID-19 revealed statistically significant differences between the years of service and the degree to which the participants felt discomfort caused by the protective equipment F(6, 152) = 3.729, *p* < 0.05, the degree of the feeling of anxiety and difficulties that are related to transmission and infection control F(6, 152) = 3.093, *p* < 0.05 and the feeling of burden in patient care F(6, 152) = 7.868, *p* < 0.05. The hypothesis tested was that means were equal across all groups of years of previous service as a nurse versus the alternative that at least one mean differed from the others. More specifically, participants with 11–15 years of experience felt more discomfort caused by protective equipment, compared to those with 26–30 years of experience.

### 3.4. Mental resilience scale

While the mental resilience of the participants was moderate (*Mean* = 19.8, *SD* = 3.06), the study revealed that from the t-test between the gender of the participants and the mental resilience scale, there was no difference between gender and the scale of mental resilience, since the significance level was greater than 0.05. In this study, there was a difference between the experience of caring for SARS-CoV-2 patients of the participants and the mental resilience scale, due to the level of significance (*t*151 = 2.373, *p* < 0.05). More specifically, participants who had experience caring for SARS-CoV-2 patients had higher mental resilience than those who did not have experience caring for SARS-CoV-2 patients.

ANOVA test between the participants' time shift and the mental resilience scale presented a difference between the shift with the hours of the participants and the mental resilience scale F (3, 152) = 4.914, *p* < 0.05. The hypothesis tested was that means were equal across all time shifts versus the alternative that at least one mean differed from the others. More specifically, participants with a cyclic schedule had lower mental resilience than morning shift workers.

### 3.5. Strategies for coping with stress during the COVID-19 outbreak

The research participants stated that they use little stress coping strategies during the period of the COVID-19 pandemic (*Mean* = 68.75, *SD* = 11.664). There was a difference between the gender of the participants and the coping strategies for stress during the period of the COVID-19 pandemic (*t*151 = -2.644, *p* < 0.05). More specifically, women used stress coping strategies to a greater extent during the COVID-19 pandemic, compared to men. Furthermore, there was a difference between the family status of the participants and the coping strategies during the period of the COVID-19 pandemic F (4, 152) = 5.242, *p* < 0.05. More specifically, the participants who were single applied to a lesser extent the strategies to cope with stress during the period of the COVID-19 pandemic, compared to the married ones.

There was, also, a difference between the participants' having children and their coping strategies during the COVID-19 pandemic (*t*151 = -3.029, *p* < 0.05). More specifically, participants who did not have children used stress coping strategies to a greater extent during the COVID-19 pandemic, compared to those who had not. T-test between having experience caring for SARS-CoV-2 patients and stress coping strategies during the COVID-19 pandemic revealed a difference between having experience in caring for SARS-CoV2 patients and coping strategies during the COVID-19 pandemic (*t*30.9 = -2.422, *p* < 0.05).

### 3.6. Relationship between the main variables

From the Pearson correlation test between the main variables (H_0_: r = 0 vs H_1_: r ≠ 0), it was observed that there was a moderate relationship between the job satisfaction of the participants and the consequences experienced by the health professionals of the reference hospitals COVID-19. Also, there was a minimal relationship between work-related burnout and the stress of the participants, the brief resilience scale along with the coping strategies of stress during the period of the COVID-19 pandemic. That is, as the work-related burnout of the participants was increased, so did the consequences experienced by the health professionals of the reference hospitals of COVID-19 (stress, mental resilience and coping strategies during the period of the COVID-19 outbreak).

### 3.7. Factors affecting the burnout of ICU COVID-19 nurses

To investigate the factors influencing occupational burnout, a multiple regression test was conducted between the participants' total burnout and the consequences experienced by health professionals of the reference hospitals of COVID-19, stress, mental resilience and coping strategies, at the 0.05 significance level. As the dependent variable, participants' total burnout score was used, and as independent variables, consequences experienced by health professionals of the reference hospitals of COVID-19, stress, mental resilience and coping strategies were used (model equation: Y = a+b_1_*X_1_+b_2_*X_2_+b_3_*X_3_+b_4_*X_4_+error). Even though the coefficient of determination of the model was relatively low and equal to 0.225, it was statistically significant F (4,152) = 10.742, *p* < 0.05. These. When the association of the independent variables was checked (H_0_: b = 0 *vs*. H_1_: b ≠ 0), only consequences experienced by the healthcare professionals of the reference hospitals of COVID-19 was found to be significantly associated with total burnout of the participants (b_1_ = 0.199, *p* < 0.01). The other factors did not affect the overall burnout of the participants because the level of significance was greater than 0.05, see Table 6.

**Table 6.** Regression analysis results with CBI total score as dependent variable.

Model	Unstandardized Coefficients		Standardized Coefficients	*t*	Sig
	b	Std.Error	Beta		
1 (Constant)	-0.329	10.592		-0.031	0.975
Resilience Scale	0.270	0.363	056	0.745	0.458
Coping strategies during COVID-19 pandemic	0.150	0.097	119	1.555	0.122
Stress	0.015	0.030	039	0.513	0.608
Consequences	0.199	0.039	402	5.144	0.000

The total burnout of the participants was affected by the consequences experienced by the healthcare professionals of the reference hospitals of COVID-19. That is, the degree of agreement of the participants on the consequences they experienced increased, and so did their burnout. The other factors did not affect the overall burnout of the participants because the level of significance was greater than 0.05.

## 4. Discussion

Most of the participants were female, were married, had 2 children, worked in rotating shifts and had an ICU experience of 1–5 years, while most of them had experience in caring for SARS-CoV-2 patients. Regarding our participants' burnout, moderate levels have been recorded, considering the total Mean score of the CBI scale. This finding is in contrast with the results of Alsulimani et al. that concluded a high degree of burnout among healthcare providers in Saudi Arabia [28]. The moderate degree of burnout among nurses in the present study may be related to nurses' coping and gradual psychological adaptation after two years of the pandemic. Gradual psychological adaptation was, also, noted during the SARS-CoV-2 period [29]. According to Carayon & Gurses [30] and Edwards et al. [31], nurses reported average to a low degree of burnout while they were working in high stress health care environments. A higher mean score was recorded for work-related and personal burnout, while patient-related burnout represented a lower Mean in the present study. Similar results have been recorded in other studies [28],[32],[33]. The distinguishment between personal and work-related burnout is indicative of individuals who feel exhausted due to external factors such as health problems or family difficulties.

Nurses' burnout was statistically significantly increased when they experienced a greater burden related to patients' care, more stress because of the danger of disease transmission and infection, more discomfort caused by using personal protective equipment to protect themselves and others from being exposed to the infection, more preoccupation and anxiety due to COVID-19 consequences on nurses' personal and professional lives and the prevailing daily work and social conditions during the pandemic. Similar findings have been recorded in the study of Zhang et al [34], who emphasized the effect of uncertainty about the duration of the current working conditions, fears in case of infection and skin damage caused by prolonged wearing of protective equipment on the emotional state of frontline nurses in China. During the pandemic, nurses had to make tough decisions, considering the best interests of patients lacking decision-making capacity and without the presence of relatives [35]. Fear experienced by nurses caring for patients with COVID-19 could lead to fatigue and discomfort, as well as a feeling of helplessness [36]. The perceived threat of exposure to COVID-19 was significantly related to burnout [37].

Having to deal with death issues, inadequate preparation in order to handle patients and their families' emotional needs, discrimination, workload, uncertainty of a therapeutic outcome and conflicts with nursing staff, physicians, superiors or patients and their families caused stress to nurses of our sample. Having to deal with patients dying alone can increase death anxiety in nurses and deteriorate nursing care quality in critical circumstances [38]. Nurses' exposure to critical and end-of-life patients, their increased responsibilities and heavy burden combined with insufficient skills in order to rapidly respond may lead to higher occupational stress, which could make nurses more vulnerable to burnout [39],[40].

Seeking professional psychological help has been associated with more perceived stress caused by the above factors. Additionally, nurses who reported greater COVID-19 consequences on their personal and professional life, greater burden related to patients' care, greater stress because of the danger of disease transmission and infection and greater discomfort caused by using the protective equipment sought professional psychological help. Finally, it has to be noted that nurses who worked in hospitals with no psychological support groups and never sought any kind of psychological help during the COVID-19 pandemic experienced higher total burnout, as well as higher personal, work-related and patient-related burnout. Consequently, the importance of providing psychological support and counseling for nurses is emphasized, given the fact that only a minority had sought psychological support inside or outside their workplace during the pandemic period.

Female nurses and nurses with children reported higher total burnout, as well as higher personal, work-related and patient-related burnout. Female gender and being a parent have been associated with higher levels of the aforementioned types of burnout. The majority of the participants were women, as the nursing profession is mostly represented by the female gender. Higher levels of burnout among female nurses have been reported in several studies [41],[42]. The nursing profession and daily clinical practice could be very stressful, even outside the pandemic context [43]. Female nurses try to balance work demands and domestic difficulties (i.e., house or children responsibilities) [44] and are more emotionally involved in workplace relationships [45],[46]. Considering the fact that the female gender has been significantly associated with anxiety and depressive symptoms in several previous studies [47],[48], our finding related to women being more vulnerable to burnout could be explained. Furthermore, it is socially acceptable for women to express their feelings, whereas men have learned to hide their emotions [49],[50], which may, partly, explain the higher burnout reported by female nurses.

Additionally, it is socially acceptable for women to care, nurture and show concern for other people, whereas men are more likely to distance themselves psychologically from patients under stressful conditions. It should be noted that female nurses felt a greater burden related to patients' care and more discomfort caused by protective equipment, compared to male nurses in the present study. Prolonged wearing could cause traces of scars on female nurses' faces more easily. Also, wearing a face mask makes nurses' face to face and eye contact with patients extremely difficult [51], which could complicate their relationships with them, contributing to higher patient-related burnout. Higher patient-related burnout could, also, be justified by nurses' fear of being infected and putting family members and especially their children at risk [52], which could be the main cause of our female nurses' burnout. All these reasons could explain the fact that female nurses in our sample used statistically significantly more stress coping strategies during the COVID-19 pandemic, compared to male nurses. Consequently, female nurses should have easy access to psychological services provided in their workplace, especially during periods of urgent and critical conditions.

More specifically, higher perceived stress among nurses with children has been reported due to their coping with death issues, preoccupation and worry about COVID-19 consequences to their personal and social life. Similarly, higher levels of stress have been noted in the study of Arafa et al. [53] regarding nurses who lived with children and older adults. Worries about children among nurses who have been separated from their children for a while in the COVID-19 pandemic have been reported by Co et al. [54] due to their fear and stress of infecting their family members [55],[56]. The fear of infecting their family was associated with an increased feeling of loneliness [34],[57]. A feeling of loneliness could, also, be experienced, perhaps, even more intensely by nurses who had no children, and this could be the reason why they have used statistically significantly more stress coping strategies during the COVID-19 pandemic.

Married nurses experienced statistically significant higher personal and total burnout in the present study. As previously mentioned, married nurses could have more anxiety symptoms because of their fear of infecting their families [58]. The higher personal and total burnout may be explained by the higher perceived stress among married nurses of our sample due to conflicts with the nursing staff and physicians, heavy responsibilities related to nursing care of these patients and preoccupation and worry about COVID-19 consequences to their personal and social life outside their workplace. Family responsibilities and the variety of work difficulties could be the cause of increased levels of burnout [52],[59]. As a result, this could justify the finding that married nurses used statistically significant more stress coping strategies during the COVID-19 pandemic.

Nurses with 11–15 years of work experience reported greater personal, work, patient-related and total burnout compared to those with 26–30 years, as they felt more discomfort caused by using the protective equipment and more stress because of the danger of disease transmission and infection. Nurses with longer work experience may be less likely to develop burnout as they may be more professionally mature, more capable in using emotion regulation and adequate coping strategies, and more effective in managing their relationship with patients and making fewer errors in critical situations [60]–[63]. This could explain the reason why nurses with more working experience used statistically significantly less stress coping strategies during the COVID-19 pandemic in this study. Additionally, nurses with 26–30 years may be more compassionate due to greater personal experience of being ill [64], greater empathy, more flexible work schedules and higher monthly salary [65]. On the other hand, nurses with 11–15 years of work experience reported greater personal, work, patient-related and total burnout than those with 1–5 years. Younger nurses at the beginning of their professional life may be less committed to work, as they have more part-time contracts compared to their older colleagues, which could contribute to the lower levels of burnout [66].

Higher academic degrees were associated with decreased stress related to the aforementioned factors. Additionally, nurses with higher educational levels seemed to be less preoccupied and anxious as far as COVID-19 consequences to their professional and personal life are concerned. This result could possibly be explained considering that higher education may lead to more sufficient training in the management of work problems and greater abilities, and while feeling confident, they may experience a greater sense of power and mastery over their work and personal life challenges and tasks [67].

The majority of our study nurses presented moderate levels of resilience. Significant determinants of resilience included previous experience (participants who had previous experience with providing nursing care to patients with SARS-CoV-2 were more resilient compared to those who had no such previous experience), working in circadian shifts (nurses working in rotating shifts were less resilient when compared with nurses working day shifts) and seeking psychological help (nurses who had sought psychological help were more resilient compared to those who had never sought any kind of psychological help). According to the findings of the present study, participants who had received training in caring for patients with SARS-CoV-2, mostly agreed with being more prepared for the pandemic, and nurses who had provided care to patients with SARS-CoV-2 used statistically significantly less stress coping strategies during the COVID-19 pandemic, compared to nursing staff with no previous experience with SARS-CoV-2. The aforementioned finding could be indicative of higher resilience. Previous experience in caring for infectious patients may also contribute to increased knowledge. This could be the reason for those nurses being more anxious about the uncertainty of the therapeutic outcome and the occurrence of discrimination compared to nurses with no previous experience. Lower resilience of nurses working in rotating shifts may result from their difficulties participating in social life activities and responding to their domestic responsibilities. Social and organizational professional psychological support could help nurses in adopting more positive emotions and attitudes, reducing their anxiety levels and consequently improving their resilience levels [55]. These findings pointed to the necessity to develop and apply interventions to improve the psychological support provided to nurses, especially during these stressful periods.

## 5. Limitations

Regarding limitations of the study, the cross-sectional method does not allow the establishment of causality between variables. Self-reported data collection and using a convenience sample also limits the generalizability of the results, which may offer a starting point for further investigation of burnout in Greek nurses. Last but not least, electronically distributed questionnaires were dispersed mainly to nurses after their agreement for this study. This situation had a negative impact on direct communication with the healthcare staff.

## 6. Conclusions

The findings of the present study confirmed moderate levels of burnout in Greek ICU nurses during the COVID-19 outbreak. Burnout could be related to various professional, individual and social factors in the field of ICUs. Considering that the ICU is a highly demanding and intense work environment even before the COVID-19 pandemic, early recognition of burnout for ICU nurses is always a challenge for the administrations of nursing institutions. In order to provide individualized and high-quality healthcare to patients and their families, nursing administrations must take the most appropriate steps to improve working conditions, promote front-line nurses' well-being while dealing with protracted stressful conditions, and facilitate nurses in their workplace. By doing so, they will be better able to adapt to the sudden and extreme demands of upcoming pandemics. It will be essential for governments to develop specific business plans and healthcare policies about possible future pandemics in order to protect caregivers from extensive stress and burnout appearance and educate them toward resilience and coping with similar situations.

## Use of AI tools declaration

The authors declare they have not used artificial intelligence (AI) tools in the creation of this article.
